# Account of the Cholera in Columbia

**Published:** 1855-07

**Authors:** B. T. Heber Jackson


*Account of the Asiatic Cholera, as it prevailed in Columbia, Lancaster County, Pa., in the Autumn of* 1854. By T. HEBER JACKSON, M. D.

It is only by carefully observing and recording the conditions under which epidemic cholera prevails, that we can hope to discover its cause, and the laws by which it is governed. With the hope of contributing some materials for this purpose, we have drawn up the following report:—

It is no easy matter to follow distinctly the progress of an epidemic when it prevails extensively in a large and populous city; but in a small town, its origin and progress can be more readily traced, and we shall endeavor to do this in regard to the disease as it occurred in the town of Columbia, Lancaster County, Pa., during the month of September, 1854.

It will be useful to consider, briefly at least, the situation and aspect of Columbia, and all such conditions as might reasonably be supposed to have exerted an influence upon the development and propagation of the epidemic in that town. To this end, the reporter avails himself of a communication from Dr. A. Clarkson Smith, of Columbia.

"Columbia is situated on the north bank of the Susquehanna, forty miles above the Chesapeake Bay, and is the terminus of two canals and three railroads. The town lies on a gentle slope inclining to the northwest. Just above the town, high, bluff hills jut out into the channel of the river; while below, another ridge, running parallel with the one above, separates it from the town of Washington. North and northeast there is a valley of the most fertile land in the country. Our population is made up of representatives of almost all nations. The German and Irish, however, compose the principal portion of the foreign population. Of the latter, there are many. The railroads, canals, coal-yards, and commission warehouses give employment to so many persons of this kind, that, in their vicinity, they generally compose the bulk of the population. As a general rule, these persons drink freely of alcoholic stimulants."

The river flows with a gentle current, and for a considerable distance above and below the town is quite shallow. The surface is studded with innumerable islets covered with a rich vegetation, which adds much to the beauty of the scene, though not, perhaps, to the salubrity of the locality. Above Washington, and a short distance below the town, a dam has been constructed for the purpose of backing the water into the canal. This structure is provided with an outlet or sluice. Above the dam—in that section of the town bordering upon the river, and west of north—is the "basin," which empties its contents, by means of the outlet-lock, into the river. Between the dam and the outlet-lock are the "reservoirs" from whence the hydrants are supplied. The condition of the river, the basin, and hydrant-water will engage our attention when we come to investigate the possible causes of the appearance of cholera in Columbia. "For several weeks previous to the appearance of cholera, remittent fever had been unusually prevalent. It began generally with bilious diarrhœa, or when this looseness of the bowels was not found, there was an extraordinary susceptibility to the action of cathartic medicine. The great prevalence of remittent and intermittent fever had left many persons debilitated, and in a condition the most susceptible to the action of any epidemic influence which might chance to come upon them.—As a general rule, persons thus debilitated were *first attacked* by cholera." Some weeks previous to the appearance of cholera as an epidemic in Columbia, the inmates of a house on Front street, not far from the place of its re-appearance, were attacked by the disease in its most fatal form, and several fell victims to its violence. This building was destroyed by order of the town authorities, and no new cases occurred until Wednesday, the 6th of September. "At this time," writes Dr. Smith, "the emigrant train, west, left at our depot two German emigrants, sick with cholera.—One of them died during the night; the other, a boy, seemed to recover from it, but afterwards died with what, I have no doubt, was ship fever; the vessel in which they crossed the Atlantic having had that disease, with cholera, on board. On Friday evening two cases occurred among our own citizens, both of whom were unhealthy men; one of these having suffered with diarrhœa for two weeks previously. At midnight, it made its appearance in almost every section of the town, and at daylight there were *thirty cases*, all of which proved fatal during Saturday. Of those who fell under my care, I recognized many whom I had noticed in the room with the German emigrants during their illness. Most, if not all of these persons had diarrhœa the day previous, but as there was nothing to alarm them, paid no attention to it until too late. Most of them were sinking into collapse when first seen by the physicians."

The class of the community chiefly affected by the disease was composed of German laborers. But on this fatal night *all* portions of the town, *all* classes of persons were compelled impartially to contribute victims to the merciless pestilence. By referring to the accompanying plan of the town, this will clearly appear. A panic now seized upon the citizens, and many of those whose means enabled them to leave, fled from the devoted town. By Monday, it is calculated that more than half of a population, numbering some 5,000, had left, and numerous persons left daily, until the week was far advanced. It is necessary to mention this, lest the erroneous conclusion might be drawn, that the working class was much more obnoxious to the disease than they really were. It is fair to presume, from the history of the epidemic during Friday night and Saturday, that, had all the citizens remained, no distinction of class would have availed as a protection, but all would have suffered alike, in proportion to their numbers. Upon those whose health was vitiated by intemperance, and the many privations attendant upon poverty, the arrows of pestilence might be expected to fall with fatal effect; but it did not appear, on Friday night and the succeeding day, that these conditions served to invite or determine the direction of their flight.

It is proper, now, to investigate the cause of the appearance of cholera in Columbia. This town is peculiarly liable to those morbific influences which are supposed to generate malarious diseases. Accordingly, we find the physicians of the place report the almost constant prevalence amongst them of intermittent and remittent fever, and, did the statement need confirmation, it is to be found in the appearance of the inhabitants. An unusual continuance of dry and very warm weather had lessened the volume of the river to a degree unknown for years; had exposed and promoted the decomposition of the vegetable matter on its banks and islets, and diminished the rapidity of its current. And, in addition, it is stated in a Columbia paper that "the canal had been made the receptacle for animals dying in the cars while being transported to the eastern markets, thrown in during the night, and washed to the shores to putrefy in the blazing sun." The sluice being closed by direction of the Tide-Water Canal Co., the escape of the double products of animal and vegetable decomposition was most effectually prevented. Upon the sluggish waters of the river, therefore, and upon its banks and islets were to be found, in even greater than usual abundance, the noxious properties, which, co-operating with a uniformly high temperature, are so generally supposed to generate malarious diseases. And there were very many sick with bilious fever, intermittent and remittent; and there were likewise many cases of bilious dysentery and diarrhœa. This might have been anticipated. The history of this year, so far, differed in no essential respect from that of former years. For the same causes, real or apparent only, had always been present at this season of the year, in a state of greater or less intensity, and like diseases, as effects, attended. In degree, as in kind, effect answered to cause; for the condition of the river and its precincts being such as to afford more than the usual amount of miasmata—"for several weeks previous to the appearance of Cholera, remittent fever had been *unusually* prevalent." Cases of diseases mentioned, continued to occur up to Friday, when the cholera broke out; they then disappeared, and re-appeared only when the cholera epidemic was abating.

In former years, the diseases peculiar to the several seasons were impressed with the leading characteristics of the malarious fevers; this was true in regard to pneumonia, functional derangements, and organic lesions of the stomach and bowels, and also entero-mesenteric fever. But now, cholera has entirely superceded the malarious fevers, and is, by no means, impressed with their characteristics. The two diseases are essentially different, and will hold no intercommunication. Are they, then, effects of the same cause? The hygrometic and thermometric condition of the atmosphere continued the same; the state of the river, of its putrid burden and noisome shores, was unaltered—at least there was no apparent change; and yet the usual and concomitant fevers have suddenly disappeared, and, in their stead, is this strange pestilence! Some were satisfied to assign miasmata as a sufficient cause. Strength, though not actual confirmation, will be lent to this hypothesis by a knowledge of the following phenomenon: On Friday, about noon, a southeast wind began to blow—of course, blowing over the river directly into the town. On the afternoon of this day the first case of cholera occurred. This wind prevailed steadily until Sunday morning, when it changed to the northwest, blowing in a direction directly from the town to the river. A very sensible diminution in the number of new cases was observed at once, and when the wind veered to the original quarter (southeast,) as sensible an increase was perceived. This curious coincidence (?) was noticed at the time by several. Indeed, it was a matter of general remark, and the "direction of the wind" was being constantly questioned with marks of deep interest and solicitude. But remarkable as it may appear, before it is determined that emanations from the river, wafted into the town by this southeast wind, were productive of the cholera, it will be worth while to remember that during a long series of years Columbia had been exposed to precisely the same influences, the same combination of circumstances, and yet remained happily free from cholera. It is not denied that the condition of the river air, probably impure, may have afforded a suitable nidus for the disease, and that the wind, by wafting the exhalations—the blastema, so to speak—into the town, may have thus exerted a powerful agency in the advancement of the epidemic. Moreover, if the river and its shores are to be accused of having generated the cholera poison, why and how did the people of Wrightsville, on the opposite bank from Columbia, escape, especially when, as on Sunday, the strong northerly wind was blowing? And yet escape they did without a single case. In addition to this, it is to be observed that Washington, on the same side of the river with Columbia, and distant only one or two miles, also escaped. When the cholera had disappeared from Columbia, a few cases were reported in Washington.

It may be said that Washington was below, and separated from the area of the peculiar miasmata, by the dam. But few would care to repose upon such a protection. We have already described the position of the basin and canal; by some it was contended that their impure waters mingling with the river water, and being then pumped up into the reservoirs, and used by the inhabitants for drinking, and for culinary purposes, were alone concerned in generating the epidemic. But it was well known to all the physicians that very many who confined themselves, from habit, to the use of pump water, were attacked with fatal effect. This is surely a sufficient refutation. Perhaps the most popular explanation was that which attributed the appearance of the malady to contagion. There is much to be said both for and against this hypothesis. In favor of the affirmative it may be urged that the appearance of the epidemic was subsequent to the arrival of the two emigrants, one of whom died with the disease; that many of those who were in attendance upon these aliens were amongst the first to be attacked; and, lastly, that many cases can be instanced, in which owing to peculiar circumstances, the disease *could* have been contracted in no other conceivable way than by contagion.

The first proposition, that of the sequence of the local epidemic upon the arrival of the imported cases, is true enough. Unless taken, however, in conjunction with, and supported by other and more weighty facts, it is really worth nothing; for it is obviously unfair to conclude that, because the cholera appeared after, it must have depended for its existence upon the arrival of the emigrants. But this argument for contagion acquires importance when taken in connection with the next fact, viz: that many of those attacked had communicated directly with the sick emigrants. "Of those who fell under my care," says Dr. Smith, "I recognized many whom I had noticed in the room with the German emigrants." But "at *midnight* the cholera made its appearance in *almost every section* of the town, and by daylight there were *thirty cases*, all of which proved fatal during the same day." Now, amongst these thirty cases, what more likely than that some, or even "many of those seen in the room with the German emigrants," should have been found? Doubtless, there were many more drawn by curiosity, or more laudable motives, about the emigrants who were *not* attacked. But, above all, it is worthy of notice, that cases of Asiatic cholera had occurred in Columbia previous to the arrival of the emigrants; and if contagious *now*, the disease did not appear to be so at that time.

After the arrival of the reporter in Columbia, on Sunday the 10th, he neither heard of, nor was himself conversant with any case that could be fairly traced to contagion. Of all the physicians (except the much to be lamented Dr. Cochran, who was seized on the first, and died on the second day of the epidemic,) officers of the sanitary commitee, and nurses in attendance upon cholera patients in private practice, or at the Town Hall, but *one*, a nurse, was attacked! And yet all these were most zealous and constant in attendance upon the sick. The emigrant who died of cholera, died on the night of the day on which he reached Columbia; no one of the citizens were attacked before Friday evening. Is this, then, to be considered the duration of the incubative stage of the cholera, if contracted by contagion? Perhaps the period of incubation varies—for, between midnight and daylight, there were thirty cases. But did the disease spread as those known to depend upon contagion are propagated, viz: from one to another, at appreciable intervals of time? No: "at midnight it made its appearance in almost every section of the town, and by morning there were thirty cases!"

Surely, this malignant disease which thus, in a few hours, invaded the whole town, could not have been produced by contagion, which requires either actual contact, or at least the reception into the body of some emanation from an individual affected with a similar disease.

But all the thirty cases had not been in contact with the emigrants, and it is probable that not more than two or three had been so actively engaged about the persons of the sick, as were the numerous physicians and nurses, and yet but one of the latter died! Moreover, it is necessary that these poisonous emanations from the person of one laboring under a contagious disease, should not only be inhaled, or otherwise effect an entrance into the body, but that they should likewise undergo some change, in which the body participates, before it can become a new focus of contagion. This change requires time, which varies much according to the susceptibility of the individual. But, on Friday night, thirty were seized almost simultaneously.

Contagious diseases do not seize upon great numbers at once; but progress from case to case. Therefore, it does not appear that the disease was simply contagious, nor that the cause of its extensive spread was of an epidemico-contagious character. Doubtless, there was some modification of the atmosphere favorable to the extension of the disease *in the town*; but this appeared to be connected with some *local* condition, for those living in the country and near to the town, escaped altogether. In company with Dr. Smith, the reporter visited many individuals in the country adjacent to, and within a mile or two of Columbia; these were affected with various ailments, and one, a gentleman advanced in years, and liable to derangement of the bowels, appeared to have cholerine; but he readily recovered under the use of simple measures. The reporter believes this to have been the only case of the kind in the country. Communication with the country, during the first three or four days of the epidemic, was indeed very limited.

Still, physicians from the town visited their patients in the country, living near to Columbia, and yet the disease, if portable, was not conveyed to any one.

Hence, it would appear that the cause of the appearance and spread of malignant cholera in Columbia, was manifestly connected with the air and locality; that it was eminently endemico-epidemic.

Before quitting this subject, it will be proper to give some individual cases which would appear to favor the doctrine of the contagiousness of Asiatic cholera: "A gentleman from Bainbridge visited this place during the prevalence of cholera, returned home, a distance of seventeen miles, took the disease and died. His family fled, and he was left in charge of a friend who also took it, as did another who assisted in his burial. Neither of these persons had been to Columbia, nor at any place where they could have contracted it save from his person." Isolated cases of this kind by no means prove that cholera is contagious, though they serve to render it probable. They should not, however, be regarded apart, but in connection with the host of facts rising up on every side to prove the opposite, and amongst which so vast a multitude, a few eccentric phenomena might well be supposed the result of accident.—Hundreds left Columbia during the height of the epidemic; some of these died of the disease away from home; but so far as the reporter could learn, in only one or two cases did there appear to be a communication of the disease to others.

To attempt the description of a disease which has been so frequently and faithfully portrayed by able writers, that one coversant with their writings cannot fail to recognize it, would be superfluous. It will be sufficient to mention some modifications under which the disease appeared in Columbia. Premonitory symptoms were present in nearly all the cases, from periods of time varying from several days to a few hours. The advent of the disease found many laboring under bilious diarrhœa; many, also weakened by recent attacks of remittent fever; "and, as a *general rule*, persons thus debilitated were first attacked by cholera." The characteristic discharges from stomach and bowels were present in all the cases; in some, profuse; in most, however, remarkably moderate, and yielding with unusual facility to the agents employed to arrest them. But the welfare of the patient oftentimes did not appear to be promoted by their suspension, nor could a favorable prognosis be founded upon the circumstance of their being small in quantity. While thus mild in the particulars specified, the case frequently presented all the other worst symptoms of the disease, viz: a rapidly failing pulse; hurried respiration; anxious expression of countenance; altered tone of voice; increasing coldness, and diminishing resiliency of skin; great restlessness, and violent cramps. The nervous symptoms were the most prominent. Hence, the poison may be supposed to have acted directly upon the nervous system, without its usual irritant action on the mucous membrane of the alimentary canal; or else, that the effusions really took place into the bowels, but were retained. Opportunity to determine the last by actual inspection did not offer; but the physical signs failed to indicate the presence of a large amount of fluid in the bowels. The altered voice when present, was regarded as most unfavorable. The cramps were chiefly confined to the muscles of the lower extremities, but in some extended to the dorsal, abdominal, and in a few to the brachial muscles. These spasms induced extreme suffering. When reaction succeeded to the stage of collapse, it was usually attended with the greatest danger, from the liability to cerebral congestion; this was more especially the case with children, of whom many were attacked.

In the treatment of this disease—many agents of the most diverse characters—many methods, some with, some without a reason, or a good one—were tried. But, from the results, could be gleaned no argument in favor of the empirical practice. Indeed, the success of rational treatment was most gratifying—inasmuch as many were saved, who, for the lack of it, could not have recovered. In the stage of collapse, even, means were sometimes found to restore to the blood its lost elements, and cause the heart again to propel its life-giving contents to the exhausted capillaries. Such general measures as experience and observation had recommended, were adopted by the Sanitary Committee; these it is unnecessary to enumerate. By the delegates from the College of Physicians of Philadelphia, the resident and visiting physicians, a series of instructions were drawn up, and by the Sanitary Committee, printed for general distribution. A notable improvement in the health of the town followed their publication, from which, perhaps, a faithful compliance with the instructions may be inferred. (?)

It would be alike tedious and unprofitable to mention all the means and drugs employed in the treatment of the disease.

In view of the great discrepancies that exist as to the pathology and nature of the disease, that treatment was the safest, and no less successful, which was based upon indications drawn from the symptoms. If the physician was so fortunate as to see a case in its incipiency, he seldom experienced difficulty in arresting its progress. The diarrhœa, unless clearly to be traced to the presence of irritating ingesta, was met at once, and successfully with astringents, the particular article of the class used being determined by the preferences of the physician.

Opium, or some one of its preparations, was almost always used in conjunction with a mineral or vegetable astringent. Large opiate enemata were used with the greatest advantage when everything else had failed. It may be observed now that throughout the treatment of the disease, perfect rest and quietude in bed were deemed essential to success.

In large or small doses, some preparation of mercury was very generally employed, and that throughout the attack. In the first, second and third stages, to restore the secretions, especially that of the liver; in the fourth stage, that of reaction, as a sedative, and for its general alterative action.

Unfortunately, a large number of patients were, when first seen by the physician, already in a collapsed state, and the end, not far distant, was but too frequently fatal. Nevertheless, some even of the worst of these recovered under judicious management. The great object in this stage was to prevent the further loss of the serum of the blood, to allay nervous irritability, and support the system until it could react. To accomplish the first indication, the most powerful astringents were used, perfectly by injection; while, to supply the waste already sustained, ice-water in small quantities at a time was freely given, a salt of soda in small doses being added from time to time. Very large doses of opium were given in this stage, without inducing any impression whatever; when carried still further, a comatose condition was induced, which would cease in a short time, if the narcotic was discontinued.

Though the application of friction and warmth to the surface was markedly disagreeable to some, many yet seemed to derive relief from their use. These applications oftentimes seemed to lessen nervous excitement, to have a quieting, soothing effect upon the patient; and, certainly, were the most useful of all the agents employed for the relief of the cramps. These spasms were most rebellious in spite of all that could be done to control them. Bandaging failed to to afford more than temporary relief. Beef-tea, animal broths, salted to the taste of the patient, and likewise various stimulants were given to support the strength and equalize the circulation, so far as the condition of the blood would permit.

Perhaps sufficient caution is not used in the exhibition of stimulants. In some cases—those in which the blood, robbed of its serum, has become of the consistence of syrup—it is vain to think of deriving advantage from stimulants. If the stomach will tolerate the intrusion of dram after dram of alcohol, and quickly recurring doses of ammonia, what good will their presence in the stomach effect? Can they impart normal fluidity to the blood, or enlarge the caliber of the arterioles and venules? Even if they could greatly increase the force and frequency of the heart's action, which, fortunately, they cannot, would it not be dangerous to employ them? For a fluid of the consistency of that now found in the heart and large blood-vessels, cannot be forced through the capillary system, without rupture of these delicate vessels and consequent extravasation. Moreover, the blood in this condition is not fitted to fulfil the functions for which it was designed; therefore, care should first be taken to repair it, and it will then circulate readily enough. But in those cases in which the discharges have not been very large, internal and external stimulants may be used with advantage. But here something is wanted which will produce a sudden and powerful impression upon the whole system; and to this end, the reporter believes nothing will be found to surpass the hot-bath in efficacy. In conjunction with Dr. Smith, the reporter made frequent trial of this agent, and the result was highly pleasing to both. It was not until the epidemic had continued some days, that this agent was had recourse to, otherwise it would have been employed much more frequently.

"In the stage of collapse," writes Dr. Smith, "the hot-bath was more efficacious than anything I am aware of. Seven apparently desperate cases recovered after being immersed in it. One of these had been in collapse full thirty hours." The water should be raised to a temperature the highest compatible with safety. Prior to immersion, the patient should take some stimulus, whatever has proved most grateful to his stomach. On removal from the bath it would be well to repeat the excitant, while, at the same time, friction with coarse towels should be practiced, and the patient enveloped in blankets. Heated bricks, wrapped in some coarse material, which has been steeped in water, should be disposed along the person of the patient beneath the bed-clothes. For adults, it is necessary that the temperature of the bath should exceed 98° Fahr. Children having a delicate cuticle, and being much more impressible, do not need so high a temperature. The patient subjected to this treatment, more especially if a child, should be closely watched, lest re-action run too high; or, having lasted a short time, should begin to be followed by depression. This did not happen in any of the cases seen by the reporter, but it is well enough to be on our guard against the possibility of its occurrence.

It is well known that patients in the third stage of cholera, though with a cold surface, suffer extremely from hot, or even warm applications; hence the use of the hot-bath is not unattended with pain. This, though to be regretted, should not deter us from employing it when it is indicated. In children, when the disease could not be cut short, it was a comparatively easy matter to conduct them through the first three stages of the cholera; but the fourth was fraught with the greatest danger. "The difficulty in managing children in this disease was not in checking the vomiting and purging, nor even in rescuing them from collapse (a simple astringent mixture, and placing them in the hot-bath, being mostly sufficient to accomplish these purposes,) but in preventing an attack of congestion of the brain, which often proved fatal." By the early employment of topical depletives, and the alterative action of mercury and ipecacuanha, this morbid condition was sometimes overcome. The reporter believes general bloodletting was practiced in only one case, and then at so late a stage of the attack that but little hope could be reposed in any remedial measure whatsoever. When the cholera made its appearance in Washington, Dr. Smith made use of venesection, and the result is given below in a quotation from his letter to the reporter:—

"In the second or serous stage, I know of no remedy equal to the lancet; and I am only sorry I did not use it when the epidemic first made its appearance, so as thoroughly to have tested its efficacy. It was not until after the disease ceased to prevail as an epidemic that I determined to try its effects, and in five cases in which bleeding was practiced all rapidly recovered. I was called to see a young married lady, of full habit, who had had diarrhœa for forty-eight hours, and the morning I visited her had the characteristic serous discharges from the stomach and bowels, attended with most distressing cramps in the extremities. Altogether it was as severe a case of the second stage of cholera as I saw during the epidemic; and feeling assured she would die under the ordinary treatment, I determined to try the effect of bleeding. The blood was drawn in a full stream from a large orifice, and when she had lost f℥xvj, she complained of feeling faint. The vein was closed, and gr. ¼ morphiæ sulph. given. After this, she had not another cramp; the purging and vomiting ceased, and with the above-mentioned combination of soda water, she rapidly recovered. Subsequently, I bled four others with like results."

Should this report appear meagre and too general in its character, the fact that no notes were taken by any of the physicians will sufficiently account for it, as well as for deficiencies and errors, should any appear.

The reporter feels it is but just to state that he spared himself no trouble in the endeavor to obtain accurate information.—[*Amer. Jour. Med. Science.*

